# Integrative research on technology-assisted physical activity and biological aging: a review of wearable sensors, tele-exercise platforms, and aging biomarkers

**DOI:** 10.3389/fmed.2026.1778386

**Published:** 2026-04-20

**Authors:** Hongwei Zhang, Hongchao Zhang, Heming Chen, Junjie Liu

**Affiliations:** 1 Department of Pre-examination Center Imaging, Boao Yiling Life Care Center, Hainan Qionghai, China; 2 Graduate School, Harbin Sport University, Harbin, Heilongjiang, China; 3 College of Science and Technology, China Three Gorges University, Yichang, China; 4 School of Basic Medical Sciences, China Three Gorges University, Yichang, China

**Keywords:** space aging, biological aging biomarkers, epigenetic clock, senescence-associated secretory phenotype (SASP), technology-assisted physical activity, tele-exercise platforms, wearable sensors

## Abstract

As global populations age rapidly, extending healthy lifespan has become a major public health priority. Physical exercise is widely recognized as a key strategy to slow functional decline and promote healthy aging, but its effectiveness and optimal prescription likely vary across individuals and should be evaluated using objective technologies and validated biomarkers. This review summarizes recent developments in technology-assisted physical activity and examines how wearable sensors, tele-exercise platforms, and digital health applications can improve adherence and enable individualized interventions for older adults. It also discusses how biological aging biomarkersons for oldepigenetic clocks, senescence-associated secretory phenotype (SASP) markers, and organ-specific plasma proteomicss, and organsto quantify exercise-related changes in biological aging and support mechanistic interpretation. This review discusses current translational challenges and future research directions, and proposes a biomarker-informed precision exercise anti-aging framework to support healthy aging through innovative technology-assisted physical activity interventions. Specifically, we ask: (i) which technology modalities and intervention components most effectively support sustained, individualized physical activity in older adults, and (ii) which validated biological aging biomarkers can serve as actionable endpoints to quantify geroprotective effects.

## 1 Introduction

Global demographic aging is accelerating, with a rapidly increasing proportion of adults aged 60 years and older (1, 2). Projections indicate that by 2050, the number of people aged ≥ 60 years will exceed 2 billion, underscoring the urgency of developing scalable strategies to promote healthy aging and extend healthspan (3). This demographic transition has increased interest not only in extending lifespan, but also in extending healthspan, defined as the period of life lived in good health and free from major chronic disease, disability, or substantial functional limitation (4, 5). Aging is also associated with progressive deterioration in physiological functions essential for survival and reproduction, leading to increased vulnerability to chronic disease, frailty, and disability (6). Therefore, interventions that promote healthy aging and prevent age-related diseases have become central topics in geroscience and public health research.

Among the available interventions, physical activity is one of the most fundamental strategies for counteracting the adverse effects of aging (7, 8). Substantial epidemiological and clinical evidence suggests that regular exercise can slow biological aging, reduce the risk of chronic diseases such as cardiovascular disease, diabetes, and neurodegenerative disorders, and improve overall quality of life (9, 10). Physical exercise has a systemic effect not only by improving metabolic health but also by facilitating muscle strength and endurance, balancing immune activity, and upholding cognitive abilities (11, 12). Notably, exercise is associated with lower morbidity and mortality and is widely regarded as an important non-pharmacological strategy for extending healthspan (13). Nevertheless, despite the widespread availability of international health and lifestyle guidelines, a large proportion of the population still does not adhere to recommended levels of physical activity. This gap highlights the need for novel strategies to improve participation and long-term adherence (14, 15).

Falls are a major safety concern in older adults and are closely linked to declines in balance control, gait stability, and postural regulation, often leading to injury, functional limitation, and reduced independence (16). Because these determinants are modifiable, exercise programs targeting strength, balance, and mobility are widely considered important components of fall prevention; however, adequate monitoring and individualized progression are needed to ensure safety and effectiveness in aged populations (17). Recent literature continues to emphasize that fall risk is influenced by real-world biomechanical circumstances and movement patterns, supporting the value of objective assessment approaches (18). In addition, gait and posture-related alterations—particularly when accompanied by chronic pain or reduced motor control—may further compromise stability and elevate fall risk, highlighting the clinical relevance of tracking spatiotemporal gait features in older adults (19). These considerations strengthen the rationale for integrating technology-assisted monitoring (e.g., wearable-derived gait/balance metrics) with scalable exercise delivery models. Accordingly, technology-assisted physical activity interventions may help bridge the gap between evidence-based training and real-world implementation while also addressing safety needs in older adults.

With the rapid advancement of technology, new tools have emerged to support and enhance physical activity interventions (20–22). Wearable devices, tele-exercise platforms, and mobile health (mHealth) applications enable continuous and objective monitoring of physical activity while providing real-time data and personalized feedback (23, 24). These technologies have shown promise for improving exercise adherence, enabling personalized exercise selection, and supporting remote rehabilitation. Their relevance became especially apparent during the coronavirus disease 2019 (COVID-19) pandemic, when traditional in-person interventions were often limited (25, 26). As an example, wearables have been established to be effective in cardiac rehabilitation protocols, enhancing the exercise capacity and patient involvement (27). Moreover, sensor systems are increasingly being used to monitor adherence and optimize interventions for musculoskeletal and neurological disorders (28, 29). The integration of these technological tools has the potential to provide scalable, accessible, and cost-effective interventions tailored to the needs of diverse populations, including older adults and people with chronic illnesses.

The measurement of the success of physical activity intervention and its influence on biological aging require credible biomarkers that indicate the molecular and physiological alterations (30, 31). Biological aging biomarkers include epigenetic clocks based on DNA methylation, telomere length, and plasma proteomic markers, among others. These measures may reflect an individual’s functional aging status more accurately than chronological age (6, 32, 33). These biomarkers provide insight into aging processes and disease susceptibility, thereby making it possible to evaluate the molecular effects of interventions. For example, DNA methylation clocks have been used to assess the anti-aging effects of traditional medicinal products and lifestyle interventions, with epigenetic rejuvenation being associated with better functional outcomes (34). In addition, plasma proteomics has identified organ-specific aspects of aging that are relevant to healthspan and longevity, highlighting the brain and immune system as important targets for intervention (33). Such biomarkers are crucial in the advancement of precision medicine programs in aging studies because of their development and validation.

The integration of technology-assisted physical activity and biological aging biomarkers represents an emerging interdisciplinary field with substantial potential for precision healthy-aging research (35, 36). The combination of wearable sensor measures, tele-exercise platforms, and biological biomarkers may enable multidimensional evaluation of intervention effects, ranging from behavioral adherence to biological response. This integrative approach supports personalized interventions that can be tailored to individual variability in aging patterns and responsiveness to exercise, thereby maximizing health outcomes (37, 38). Additionally, it helps develop new digital biomarkers, including parameters of circadian rhythms based on accelerometry that are associated with mortality risk and healthspan, highlighting the complexity of the aging process (39). Continued progress in these fields has the potential to reshape clinical practice and population-level strategies for promoting healthy aging.

Although prior reviews have summarized technology-assisted physical activity interventions in older adults and home-based settings (e.g., scoping work on digital physical activity (PA) support) and others have discussed the validation and utility of biological aging biomarkers, evidence that connects specific digital intervention components to validated molecular aging endpoints remains fragmented. Accordingly, the central question of this review is: How can technology-supported physical activity be designed and evaluated—using validated biological aging biomarkers—to enable precision exercise strategies that promote healthy aging? To address this, we synthesize evidence across wearable sensors, tele-exercise platforms, and digital health applications, map the biomarker endpoints most relevant to exercise-mediated aging modulation (epigenetic clocks, SASP-related inflammatory/senescence markers, and organ-linked plasma proteomics), and highlight translational gaps to inform a practical “precision exercise anti-aging” framework.

This review is narrative in scope but was informed by a structured literature search. We searched PubMed/MEDLINE, Web of Science, and Scopus from January 2019 to October 2025, and supplemented database retrieval with Google Scholar and backward reference screening. Search terms combined concepts related to technology-assisted physical activity (e.g., wearable sensors, tele-exercise, mobile health, digital health applications, and artificial intelligence) with concepts related to biological aging biomarkers (e.g., epigenetic clocks, DNA methylation age, senescence-associated secretory phenotype, inflammatory cytokines, plasma proteomics, and digital biomarkers).

We included peer-reviewed English-language studies in humans that evaluated technology-supported physical activity interventions and/or validated biological aging biomarkers, together with selected translational animal studies and landmark methodological papers when directly relevant to mechanistic interpretation or biomarker validity. We excluded editorials, conference abstracts without sufficient methodological detail, studies unrelated to physical activity or biological aging measurement, and reports that did not provide interpretable outcome information.

Records were screened narratively by title/abstract and then by full text for relevance to the review question. Approximately 642 records were identified, 186 were assessed in full text, and 124 studies were retained for qualitative synthesis. We did not conduct a formal risk-of-bias assessment because the review was narrative; instead, we considered study design, endpoint validity, and translational relevance qualitatively during evidence synthesis.

The final synthesis was organized by technology modality (section 2), biomarker class (Section 3), and translational implementation considerations (Table 1). Throughout the review, we distinguish evidence derived from randomized or supervised intervention studies, observational human cohorts, and mechanistic or preclinical studies, and interpret causal inferences accordingly.

**TABLE 1:** Revised operational precision exercise anti-aging framework.

Framework step	Digital inputs	Molecular anchors	How the data interact in practice	Illustrative implementation example	Main feasibility/cost/data-integration issues	References
Baseline stratification	PA history, wearable baseline, falls risk, sleep, HR/HRV, digital literacy, preferences	One feasible baseline panel (e.g., epigenetic clock ± inflammatory markers)	Digital and molecular data jointly define initial risk, starting dose, and follow-up intensity	Research: baseline phenotyping before randomization. Clinical/community: simple wearable onboarding plus selective biomarker sampling in higher-risk users.	Up-front device and assay cost; unequal digital access; need for clinically interpretable starting rules	(42, 53, 62)
Dose-delivery monitoring	Session attendance, MVPA, exertion, recovery signals, symptoms, coaching engagement	Usually no repeated molecular sampling at this stage	Continuous digital data verify whether the prescribed exercise dose was actually delivered	Weekly remote review of adherence and safety flags; automated reminders or coach escalation when dose delivery drifts.	Missing data, adherence decay, sensor calibration, and staff time for oversight	(40, 58, 144)
Early response/non-response detection	Trends in gait, balance, sleep, recovery, and behavioral regularity	Optional interim low-burden markers when feasible	Digital trajectories help distinguish inadequate exposure from likely biological non-response	If function improves but biomarker response is absent, reassess sampling timing and confounding; if both are flat, modify dose or support mode.	False reassurance from noisy digital data; weak interoperability between app, wearable, and laboratory systems	(31, 33, 93)
Follow-up biomarker reassessment	Documented exposure history from the wearable/ platform period	Repeat epigenetic, inflammatory, or proteomic panel at the prespecified follow-up window	Biomarker change is interpreted in the context of actual exposure, adherence, and recovery patterns	Research: 8–12 week or longer trial endpoint. Clinical/community: selective repeat testing in poor responders or high-risk users.	Assay standardization, timing sensitivity, batch effects, and reimbursement/ affordability	(39, 52, 55, 60)

This revised framework makes the proposed precision exercise anti-aging model operational by showing how high-frequency digital measures can guide dose delivery, interpret biomarker change, and trigger adaptation. The examples are intended as pragmatic templates rather than fixed protocols.

## 2 Digital health for physical activity: wearables, tele-exercise, and AI

### 2.1 Wearable sensors: applications and advantages

**FIGURE 1:**
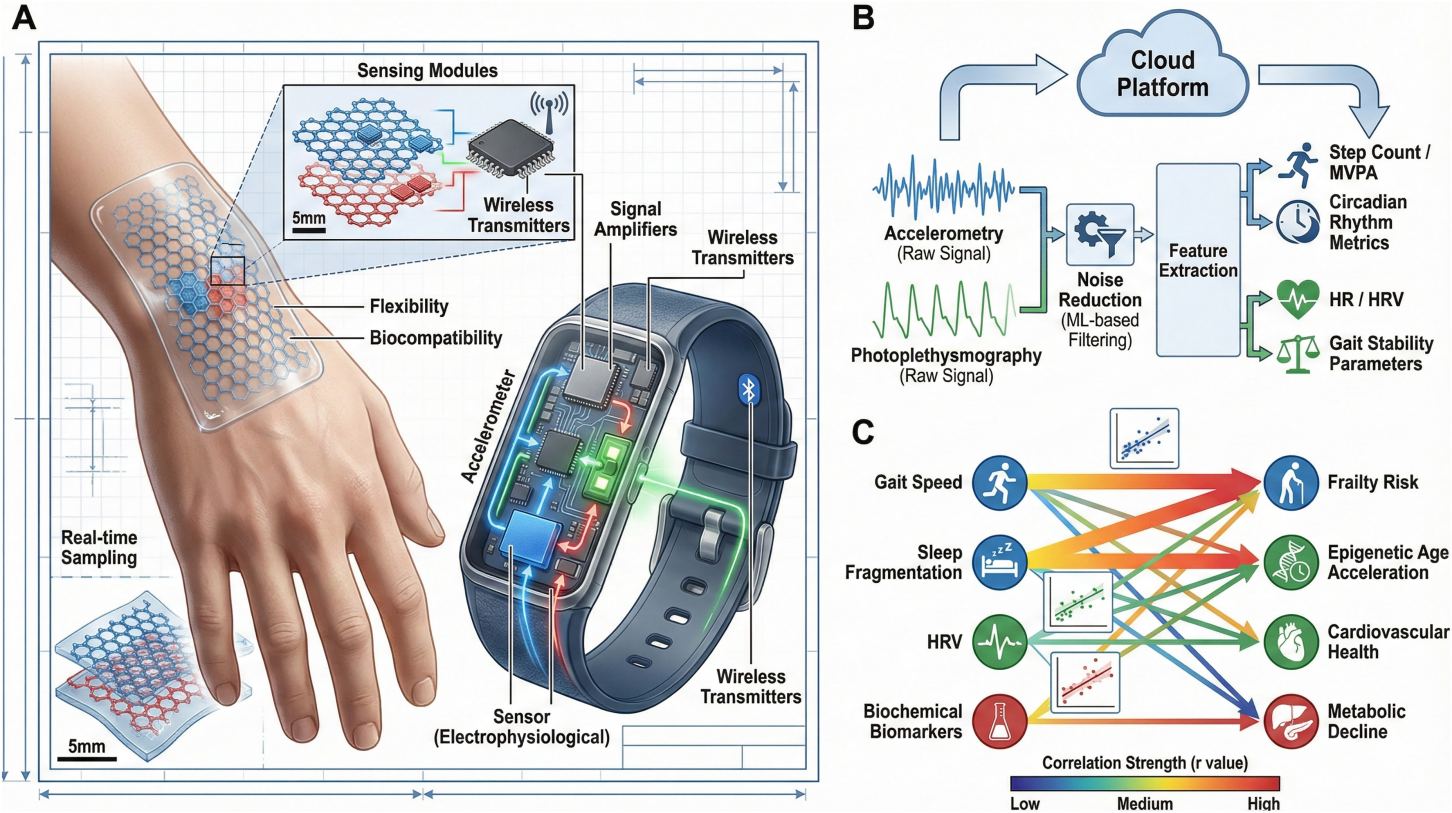
Schematic overview of multimodal wearable sensors for objective monitoring of physical activity and aging. **(A)** Advanced wearable designs and sensing modalities (For example, flexible graphene sensors and skin-like electrophysiological sensors) enabling comfortable, long-term use. **(B)** Data workflow: mechanical, electrophysiological, and biochemical signals are filtered, feature-extracted, and integrated to generate actionable metrics. **(C)** Translational relevance: sensor-derived metrics (For example, MVPA, circadian rhythm amplitude) associate with aging outcomes (frailty, epigenetic age, proteomic signatures; *r* = 0.3–0.7). Dashed lines indicate candidate digital biomarkers under validation.

Wearable sensors have become key tools for the objective assessment of physical activity because they can record multidimensional information, including movement volume, heart rate, and sleep quality, in real time (40, 41). Smart wristbands, heart rate monitors, and accelerometers are examples of devices that can continuously monitor physiological and behavioral parameters, thereby providing a detailed picture of an individual’s activity patterns (42). A recent umbrella review of 51 systematic reviews found that interventions incorporating wearable devices may increase physical activity in adults, with a median improvement of approximately 1,312 steps per day and 57.8 min per week of moderate-to-vigorous physical activity (MVPA), although the certainty of evidence was low to moderate and most included reviews were rated as having critically low methodological quality. Evidence for reducing sedentary time was inconsistent, and effects in older-adult subgroups showed greater uncertainty, underscoring the need for biomarker-embedded trials in aging populations where wearable-derived dose and adherence metrics can be linked to validated aging endpoints (43). Continuous data collection allows wearable sensors to capture behavior in real-world settings with greater objectivity than self-report methods. These data may help link physical activity exposure to functional outcomes and, in future biomarker-embedded studies, to changes in biological aging trajectories. For example, graphene-based wearable sensors offer greater flexibility and biocompatibility, improved signal quality, and enhanced capacity for continuous daily monitoring (44). Similarly, soft, skin-like wearable sensors have helped overcome earlier limitations related to device size and discomfort, thereby improving user compliance and data quality (45). Multimodal sensing (mechanical, electrophysiological, and biochemical) adds even more richness to the range of data that can be utilized in health monitoring (46). In clinical populations, such as breast cancer survivors, wearable-assisted interventions have been associated with higher levels of moderate-to-vigorous physical activity and improved weight management, highlighting the translational potential of these devices (47). Skin-mounted sensors have also been used in neurological conditions, such as Parkinson’s disease and pediatric neurological disorders, where objective assessment of motor performance and disease progression is needed (48, 49). A major strength of wearable sensors is their ability to provide unobtrusive, continuous monitoring in naturalistic settings while generating objective and quantifiable data that are often more reliable than traditional self-reported measures. These objective measurements support individualized intervention planning and facilitate the study of how physical activity influences aging processes and health outcomes (50). Despite these advantages, issues such as data privacy, sensor calibration, and user compliance remain important priorities for further research and development (51). Taken together, wearable sensors represent a promising and scalable approach for objective physical activity monitoring. However, current evidence more consistently supports their value for feasibility, adherence tracking, and functional assessment than for demonstrating direct modification of validated molecular aging biomarkers (Figure 1). Importantly, wearable sensors and digital platforms can capture continuous behavioral and physiological data during the intervals between clinic visits and biospecimen collection (52). Metrics such as daily activity volume, exercise intensity, heart-rate dynamics, sleep, recovery, and adherence may help explain why conventional biomarkers change over time and may improve attribution of biomarker shifts to actual exercise exposure rather than to self-reported activity alone. In future studies, synchronizing these longitudinal digital measures with scheduled blood-, saliva-, or other biomarker sampling may strengthen causal inference, identify early responders or non-responders, and support more precise adjustment of intervention dose (53).

### 2.2 Development and challenges of tele-exercise platforms

Tele-exercise platforms use internet-based delivery to reduce geographic barriers and expand access to supervised or guided exercise for older adults and clinical populations. These platforms became particularly important during the COVID-19 pandemic, when in-person services were limited, and they helped preserve key elements of supervision through real-time feedback, progression, and safety monitoring (48). Remote exercise interventions have been proven feasible with generally good adherence across conditions such as Parkinson disease, coronary heart disease, and among cancer survivors (54–56). The benefits of supervised exercise can also be maintained in live-remote sessions via videoconferencing while offering flexibility and convenience (57). In addition, platforms that include customized exercise regimens, often optimized by artificial intelligence (AI) models, have shown effectiveness in improving balance, strength, and flexibility in older adults without requiring laboratory-based monitoring (58). Irrespective of these encouraging results, tele-exercise platforms still face several challenges: The digital divide may constrain equitable participation (42), user interfaces must remain accessible to individuals with different levels of technological skills (59), data security and privacy require robust safeguards (60), and the lack of standardized guidelines complicates comparisons across studies (61). Overcoming such challenges will benefit from multidisciplinary teams of technologists, health care specialists, and users to optimize inclusive design and preserve data integrity (Table 2).

**TABLE 2:** Revised evidence map of technology-assisted exercise interventions and reported endpoints.

Population/setting	Technology-assisted intervention	Evidence type	Design/exposure window	Main functional or behavioral outcomes	Molecular aging/biomarker readout	Evidence strength/implication	References
Community-dwelling older adults	Smartphone motor-fitness assessment plus AI-generated personalized multicomponent exercise	Human RCT	8 weeks; 5 sessions/week	Improved balance, flexibility, and upper-limb strength in the intervention arm	No validated molecular aging biomarker collected	Moderate evidence for functional benefit; low evidence for direct aging-biomarker effects	(58)
Older rural cancer survivors	Live remote supervised tele-exercise (EnhanceFitness model)	Pilot human RCT	16 Weeks; 3 sessions/week	High attendance; improved sit-to-stand, light PA, and daily step counts	No molecular aging biomarker collected	Moderate evidence for feasibility and physical-function benefit; biomarker gap remains	(55, 57, 138)
Prefrail/frail older adults	Gaming-based tele-exercise/exergaming (CogXergaming)	Feasibility RCT	6-week supervised tele-exercise; 18 sessions	Improved dynamic balance, gait-related performance, and chair-stand outcomes	No molecular aging biomarker collected	Promising early evidence for balance-focused tele-exercise; replication still needed	(54, 56, 139)
Breast cancer survivors	Wearable-assisted physical activity interventions	Meta-analysis/meta-regression	Multiple trials; mostly short- to mid-term interventions	Increased MVPA/total PA and improved weight-control outcomes	Not designed to test biological aging endpoints	Moderate evidence for behavior change; indirect relevance to healthy aging only	(47)
Community-dwelling older adults across digital intervention trials	Mixed digital health interventions (wearables, apps, web/video-based coaching)	Systematic review	Multiple studies with heterogeneous durations and delivery modes	Generally positive effects on PA and some physical-function outcomes, but heterogeneous	Very few studies embedded validated molecular aging biomarkers	Best current evidence supports feasibility and PA promotion rather than causal biomarker change	(26, 140)

This revised table separates representative intervention evidence by study design and clarifies that most available studies support feasibility, adherence, physical activity, or functional outcomes rather than validated molecular aging endpoints. Evidence strength refers to the intervention literature for the stated outcome domain, not to proof of causal slowing of biological aging.

### 2.3 Personalized intervention potential of digital health applications

**FIGURE 2:**
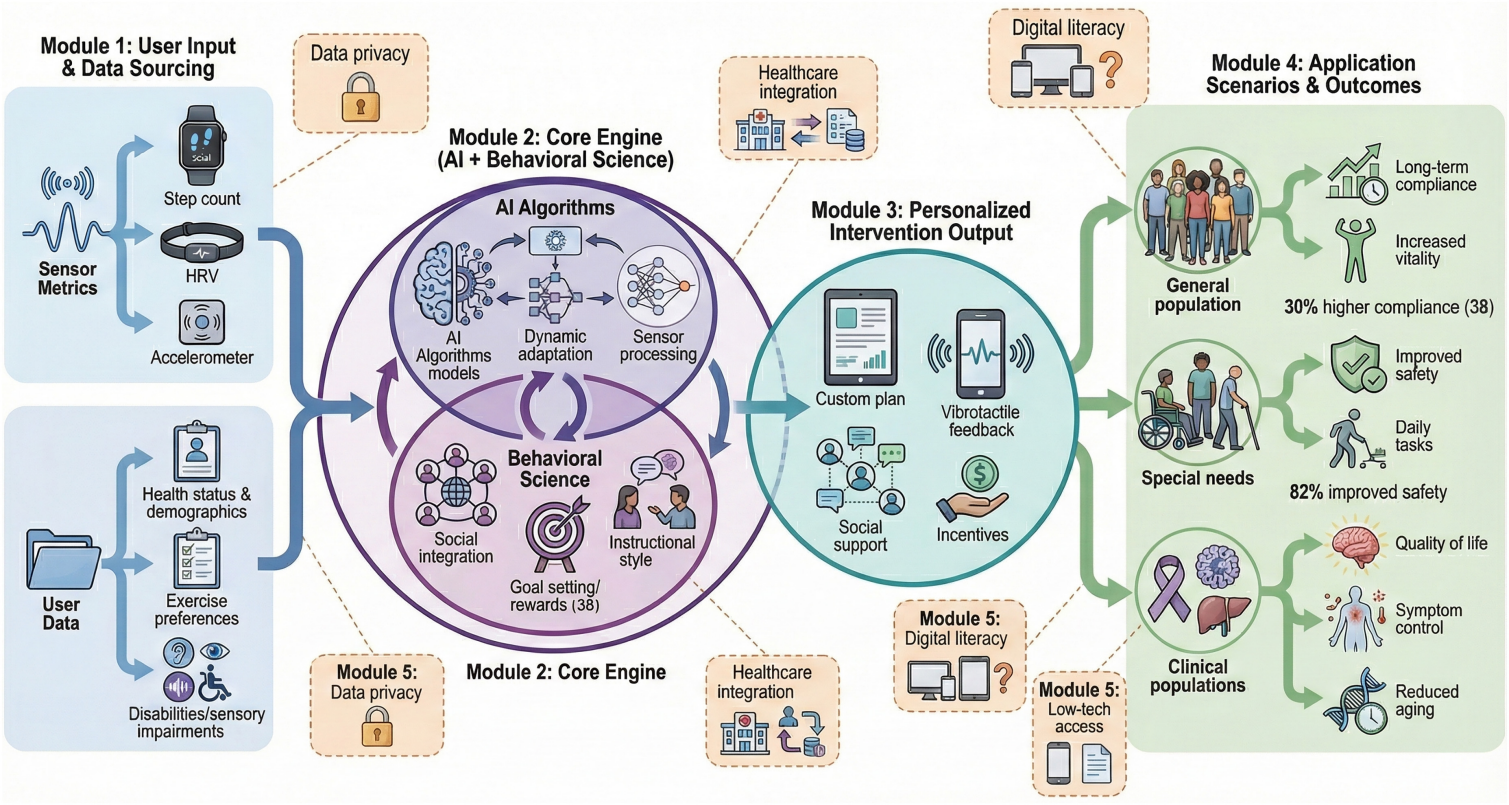
Schematic framework of DHAs for personalized physical activity interventions targeting biological aging and health outcomes. DHAs integrate AI algorithms and behavioral science to deliver dynamic, user-centric exercise interventions.

Digital health applications (DHAs) combine AI and behavioral science to provide individualized physical activity interventions that respond dynamically to individual user data (62, 63). By incorporating AI algorithms, these applications can continuously use sensor-derived metrics to adjust exercise plans and provide customized feedback that enhances motivation and training effectiveness (58). Social integration features enable interaction and support among users. These functions may improve long-term adherence to exercise programs by fostering a sense of community and accountability (42). Behavioral strategies embedded in DHAs, such as goal setting, rewards, and nudges, may also promote longer engagement and support health behavior change (42). For populations with specific needs, such as individuals with disabilities or sensory impairments, DHAs can provide multimodal feedback, such as vibrotactile or verbal cues, to improve exercise performance and safety (64) (Figure 2). Personalized DHAs have been shown to improve quality of life and symptom control in cancer survivors and in patients undergoing organ transplantation, highlighting their therapeutic value in clinical settings (55, 65). DHAs are flexible enough to accommodate different preferences in exercise type, social context, and instructional style, which may help match interventions to users’ needs and readiness levels (66). Nevertheless, challenges remain in securing user privacy and data and in integrating DHAs into existing healthcare practices, which may limit real-world implementation (60). Overall, digital health applications represent a promising pathway for delivering personalized, scalable, and engaging physical activity interventions that may influence biological aging–related pathways and clinically relevant health outcomes (Table 3). Across wearable-, tele- exercise-, and app-enabled programs, the strongest and most consistent evidence to date supports improvements in feasibility, adherence, and functional/behavioral outcomes (e.g., MVPA, mobility, and quality-of-life measures). However, as also reflected in the evidence map (Table 2), relatively few technology-assisted physical activity trials are designed to include validated molecular aging endpoints (e.g., epigenetic clocks or plasma proteomics), leaving a key translational gap between digital delivery and biological aging modification. Available studies are highly heterogeneous in their intervention components, duration, and target populations, and biomarker reporting is often secondary, underpowered, or absent, making cross-study comparisons and causal inference challenging. Future trials should pre-specify aging biomarkers as primary or key secondary outcomes, standardize sampling and analytic pipelines, and explicitly model the pathway from sensor-derived dose/adherence, physiological adaptation, biomarker change to strengthen mechanistic interpretation and clinical credibility.

**TABLE 3:** Revised comparison of technology modalities for technology-assisted physical activity.

Technology modality	Typical data streams	Primary role in exercise delivery	Current evidence base	Key limitations/implementation barriers	Biomarker-integration potential	References
Wearable sensors	Steps, MVPA, heart rate/HRV, sleep, gait and balance signals, sometimes photoplethysmography-derived measures	Objective exposure tracking, adherence monitoring, feedback, remote safety surveillance, and longitudinal behavioral phenotyping	Moderate-to-high for activity monitoring and behavior change; emerging for digital biomarker applications	Device heterogeneity, adherence decay, calibration issues in older adults, privacy/data governance, and unequal access	Can contextualize why laboratory biomarkers change between clinic visits and may support emerging wearable-based aging metrics	(39, 40, 42, 51, 53)
Tele-exercise platforms	Attendance, session duration, perceived exertion, video-observed performance, and linked wearable data when available	Remote supervision, exercise progression, continuity of care, and access expansion for home-based programs	Moderate for feasibility, adherence, and physical-function outcomes in older adults; low for validated molecular aging outcomes	Digital divide, connectivity and usability barriers, staff workload, safety escalation needs, and limited workflow integration	Useful for synchronizing dose delivery with scheduled sampling, but biomarker-embedded trials remain uncommon	(54, 55, 57, 61, 140, 141)
Digital health applications/AI-personalized coaching	Wearable feeds plus self-reports, goals, context, engagement logs, nudges, symptom check-ins, and decision-support outputs	Personalized prescription, adaptive coaching, motivational support, and automated detection of non-response or disengagement	Moderate for PA promotion and personalization; low-to-emerging for validated aging biomarker integration	Algorithm transparency, model bias, interoperability, attrition, clinician trust, and need for human oversight	Most promising for linking digital and molecular signals, but requires prespecified data models and governance	(55, 58, 62, 63, 65, 142, 143)

Evidence labels are comparative and narrative: moderate-to-high indicates support from multiple systematic reviews or meta-analyses for physical-activity or functional outcomes; moderate indicates several trials with consistent feasibility or functional findings; emerging indicates early translational potential with limited direct evidence for validated biological aging endpoints.

Despite their promise, technology-assisted physical activity interventions also have important limitations that may constrain real-world anti-aging applications. Consumer wearables differ in validity across devices, contexts, and user groups, and accuracy may decline in older adults with slow gait, altered movement patterns, arrhythmias, or assistive-device use. Engagement with digital platforms may also diminish over time, while digital exclusion related to age, health literacy, language, cost, or internet access may widen disparities in participation. In addition, tele-exercise and app-based systems generate fragmented data streams that are often difficult to integrate with clinical records and laboratory biomarker platforms. Algorithm-driven personalization further raises concerns regarding transparency, bias, accountability, and the interpretability of automated recommendations. Accordingly, these technologies should be viewed as enabling tools rather than stand-alone solutions, and their implementation requires standardized measurement, equitable design, and continued clinical oversight. As reflected in the summary tables, the current evidence base includes randomized trials, feasibility studies, observational analyses, and mechanistic work; therefore, functional and adherence benefits are supported more consistently than direct causal effects on validated molecular aging endpoints.

## 3 Exercise and biomarkers of biological aging

### 3.1 Epigenetic clocks and exercise intervention

**FIGURE 3:**
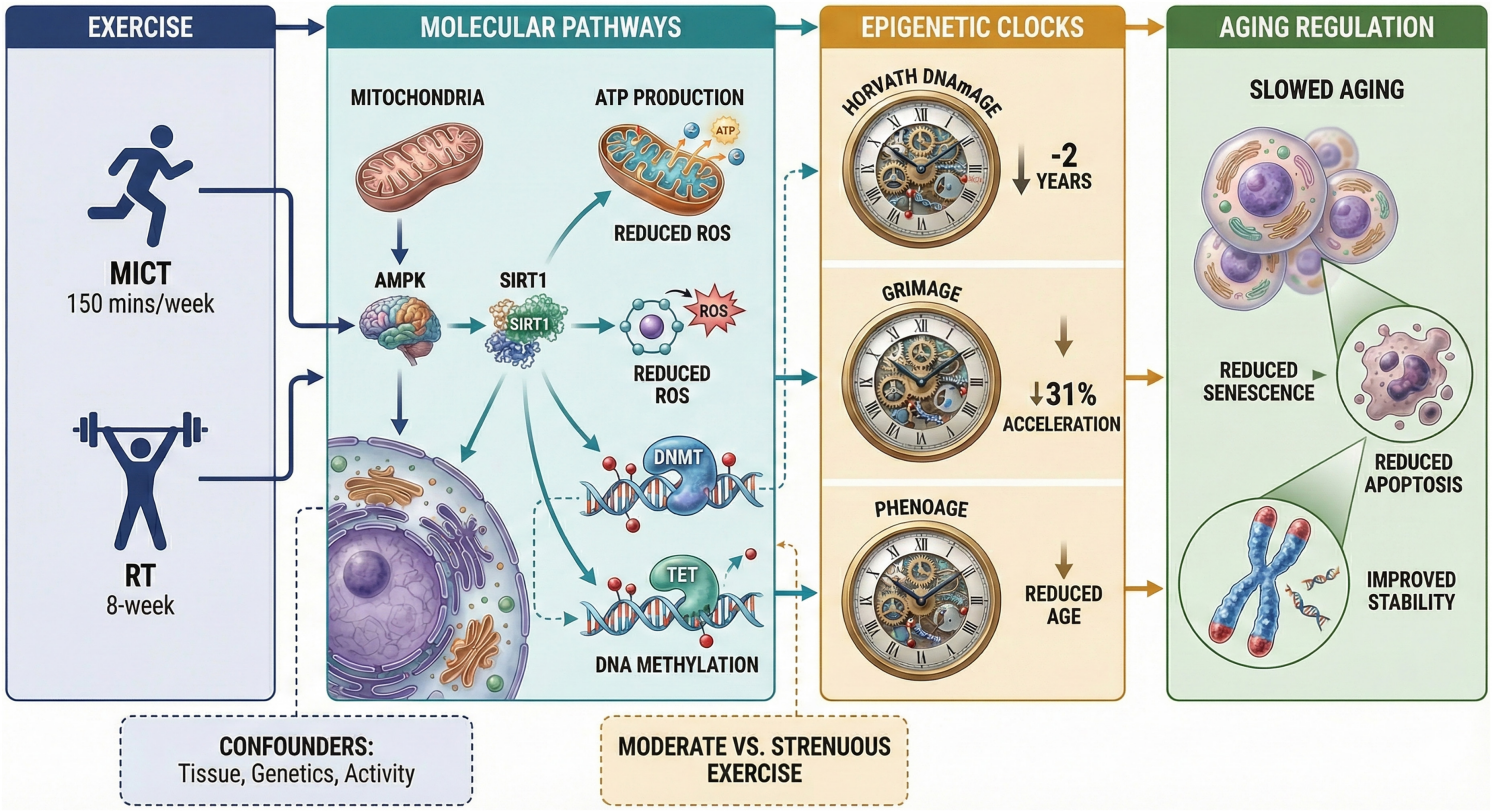
Mechanistic links between exercise and epigenetic clock regulation in biological aging. Exercise may influence epigenetic aging through interconnected pathways, including AMPK/SIRT1/PGC-1α signaling, mitochondrial remodeling, redox regulation, autophagy, and immune-metabolic signaling, thereby contributing to altered DNA methylation patterns and potentially slower aging dynamics across first-, second-, and third-generation measures, including Horvath DNAmAge, PhenoAge, GrimAge, and DunedinPACE.

Epigenetic clocks, including the Horvath clock and other DNA methylation–based age predictors, are valuable biomarkers of biological aging because they estimate age-related changes in DNA methylation patterns (67, 68). Beyond first- and second-generation epigenetic clocks, third-generation measures such as DunedinPACE estimate the pace rather than the accumulated amount of biological aging. Because DunedinPACE was developed to capture longitudinal, multisystem physiological decline, it is conceptually well suited to exercise studies that aim to detect dynamic biological adaptation. Recent evidence has further linked higher cardiorespiratory fitness to a slower DunedinPACE-defined pace of aging, suggesting that third-generation clocks may complement Horvath DNAmAge, PhenoAge, and GrimAge when evaluating the geroprotective effects of physical activity. Future technology-assisted exercise trials should therefore consider including both state-based and pace-based epigenetic measures to improve sensitivity and mechanistic interpretation (69). These epigenetic aging markers may be influenced by exercise, potentially reflecting delayed biological aging or partial epigenetic rejuvenation (70, 71). A number of studies have shown that a regular physical activity, especially moderate-intensity exercise, correlates with a marked decrease in epigenetic age acceleration. As an example, a randomized clinical trial of diet and lifestyle interventions, which included exercise, found a reduction in Horvath DNAmAge of almost 2 years in an 8-week intervention (72) (Figure 3). Likewise, observational studies in large cohorts, including the Health and Retirement Study and the UK Biobank, have reported that physically fit individuals tend to show lower epigenetic age acceleration according to second-generation clocks such as GrimAge and PhenoAge (73, 74). Mechanistically, exercise induces changes in gene expression and epigenetic regulation that may influence aging-related processes. Exercise improves the capabilities of the mitochondria and lowers the occurrence of oxidative stress, which could affect the DNA methylation patterns that can be attributed to aging. At the mechanistic level, exercise-related effects on epigenetic aging are unlikely to be explained by a single AMPK/SIRT1 axis. Rather, exercise engages a broader network that includes AMPK/SIRT1/PGC-1α signaling, mitochondrial biogenesis, autophagy, redox regulation, insulin/IGF-1-mTOR signaling, and myokine-mediated immune-metabolic communication. AMPK and SIRT1 remain important nodes within this network, but they should be presented as representative mediators rather than sole drivers of exercise-associated anti-aging effects (75, 76). Nevertheless, the effects of exercise on epigenetic clocks may differ according to tissue type, exercise intensity, duration, and genetic background. For example, acute strenuous exercise may not produce the same epigenetic effects as a controlled program of moderate exercise (77). Moreover, it has also been noted that it is important to also take into consideration the confounding variables including occupational physical activity which can have varying relationships with biological aging than leisure-time physical activity (78). Overall, current evidence suggests that regular moderate-intensity exercise is associated with slower epigenetic aging and may influence DNA methylation-based aging measures. However, causal evidence remains limited, varies by clock type and tissue, and cannot yet isolate the contribution of technology-assisted intervention components with confidence. Importantly, much of the evidence linking physical activity to lower epigenetic age acceleration comes from observational cohorts, which are informative but cannot fully establish causality or isolate the contribution of technology-supported intervention components. Interventional evidence remains comparatively limited and often involves multi-component lifestyle programs, complicating attribution to exercise dose and to digital delivery features specifically. Reported effects can also vary by tissue and clock type, and are sensitive to intervention duration, exercise intensity, and timing of biospecimen collection. These factors likely contribute to inconsistency across studies and highlight the need for adequately powered, technology-assisted randomized trials with standardized epigenetic sampling, pre-registered analyses, and longer follow-up.

### 3.2 Inflammatory cytokines and senescence-associated secretory phenotype (SASP)

**FIGURE 4:**
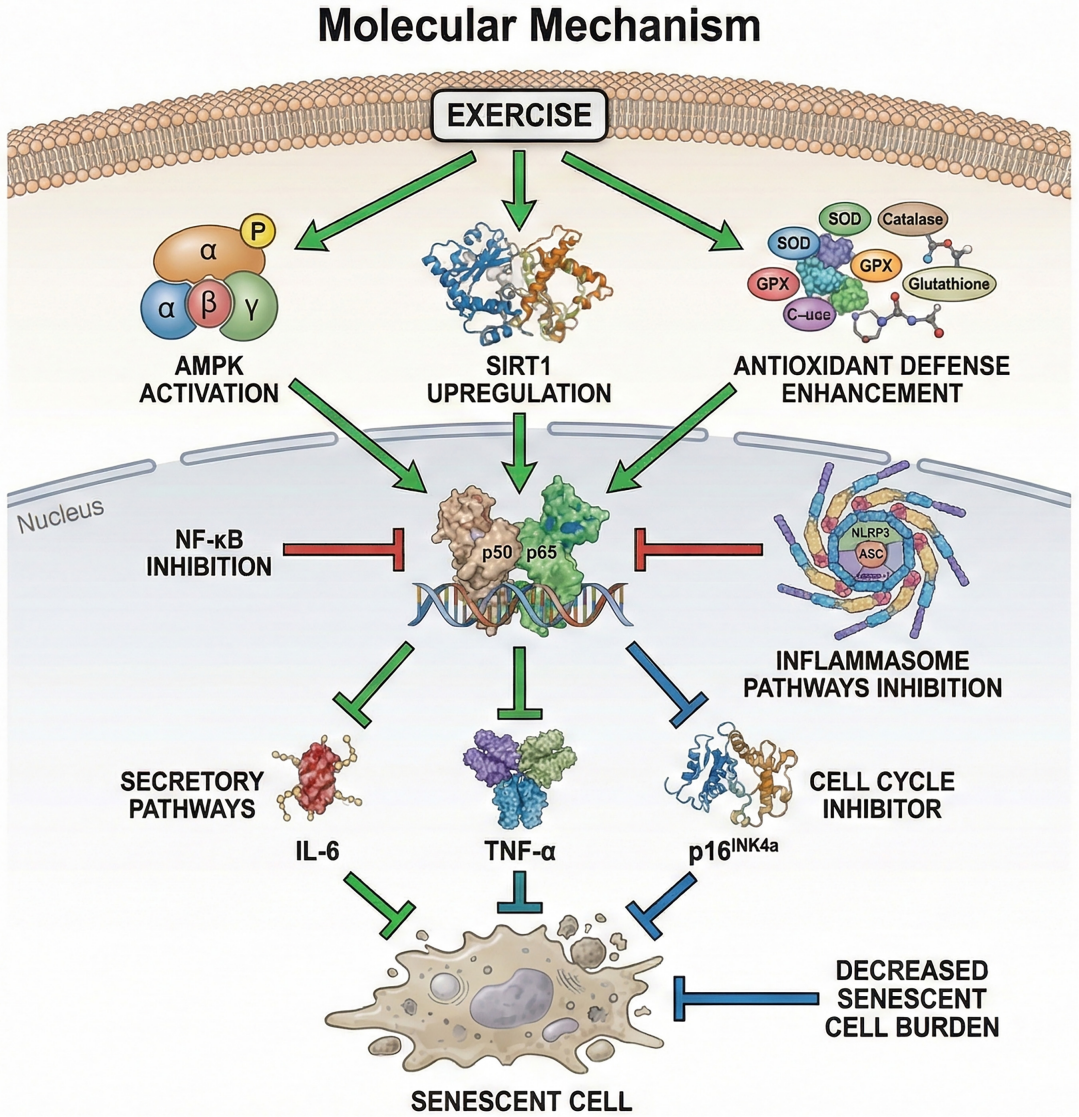
Mechanisms by which exercise modulates SASP-related inflammation and cellular senescence. Regular exercise engages multiple interacting pathways—including AMPK/SIRT1/PGC-1α signaling, redox-sensitive mechanisms, immune-cell remodeling, and NF-κB/NLRP3-related regulation—to reduce chronic SASP burden and improve tissue homeostasis over time. Importantly, transient exercise-induced release of muscle-derived IL-6 may serve adaptive myokine functions and should be distinguished from persistently elevated basal inflammatory IL-6 observed in aging and chronic disease. Moderate exercise activates AMPK/SIRT1, suppressing NF-κB translocation and NLRP3 activation, lowering SASP mediators (IL-6; TNF-α) and enhancing antioxidant defenses.

It is important to distinguish chronically elevated basal inflammatory cytokines from the transient cytokine response to an acute exercise bout. Although persistently elevated IL-6 and TNF-α are often linked to inflammaging and SASP burden, skeletal muscle also releases IL-6 acutely during exercise as a myokine, where it may support fuel mobilization, training adaptation, and, under some conditions, exercise capacity (79, 80). Accordingly, an acute post-exercise increase in IL-6 should not be interpreted as uniformly detrimental. The longer-term anti-inflammatory benefit of regular exercise is better understood as a reduction in chronic inflammatory tone and senescence burden over time, rather than simple suppression of all cytokine signaling at every time point (81, 82). Exercise-related reductions in systemic inflammation are associated with better muscle function, improved metabolic health, and a slower pace of biological aging (83, 84). Mechanistically, exercise may suppress inflammation by strengthening antioxidant defenses, reducing oxidative stress, and altering immune cell phenotype. For example, exercise may promote anti-inflammatory polarization of monocytes and reduce macrophage senescence, thereby decreasing SASP secretion (85, 86) (Figure 4). Moreover, exercise affects such molecular pathways as nuclear factor kappa B (NF-κB) and inflammasome activation, which control SASP expression (87, 88). In older adults and in patients with chronic conditions, clinical intervention studies have shown that exercise programs may reduce markers of cellular senescence and SASP-related factors, such as cyclin-dependent kinase inhibitor 2A and IL-6, and that these changes are associated with better physical function and lower frailty (89, 90). Importantly, the anti-inflammatory effects of exercise may depend on intensity: moderate exercise generally reduces inflammation, whereas high-intensity exercise may transiently increase pro-inflammatory cytokines (91). Overall, regular moderate exercise is associated with reduced chronic inflammatory tone and lower senescence-related burden over time. However, inflammatory biomarkers should be interpreted cautiously because some cytokines, particularly IL-6, may increase transiently during acute exercise as part of adaptive myokine signaling rather than harmful inflammation.

### 3.3 Organ-specific aging models and plasma proteomics

Recent advances in plasma proteomics have created new opportunities to identify organ-specific patterns of aging and to examine how exercise may modulate them (92, 93). Large-scale proteomic studies suggest that exercise remodels the plasma proteome and alters age-related proteins relevant to the heart, kidney, and brain, thereby supporting organ function and potentially slowing biological aging (78, 94). As an illustration, occupational biological age acceleration in the liver and whole body is associated with leisure-time physical activity, whereas high occupational physical activity can contribute to the heart biological age acceleration (78). Exercise induces favorable changes in plasma proteins related to inflammation, metabolism, and extracellular matrix remodeling, all of which are important for organ health (94, 95). Multi-omics analyses of proteomics and transcriptomics show that exercise alters immune signatures, mitochondrial activity and senescence indicators in skeletal muscle and other tissues that encompass systemic anti-aging effects (96, 97). In addition, exercise-induced changes in the plasma proteome have been associated with improvements in cardiorespiratory fitness and cognitive performance. These findings suggest that plasma proteins may serve as markers of exercise responsiveness and aging-related adaptation (95, 98). Organ-specific models also examine how exercise alters senescence-associated secretory phenotype factors in tissues such as the heart, brain, and kidney, thereby reducing age-related structural and functional decline (99, 100). Taken together, these findings highlight the value of plasma proteomics for assessing the organ-level anti-aging effects of exercise. They also lay the groundwork for integrated multi-organ assessment strategies to monitor the biological effects of exercise on aging more comprehensively (Table 4). Plasma proteomics offers rich mechanistic resolution, but its high dimensionality also increases susceptibility to batch effects, platform-specific biases, and multiple-testing challenges, which can limit reproducibility if not rigorously controlled. Moreover, much of the current evidence reflects exercise training effects broadly rather than trials explicitly testing technology-assisted exercise delivery with proteomic aging signatures as prespecified endpoints. Replication across cohorts and harmonized analytic workflows will be critical for translating proteomic signatures into actionable endpoints within precision, technology-supported exercise programs.

**TABLE 4:** Revised comparison of biological aging biomarkers discussed in Section 3.

Biomarker class	Representative measures	Sample/modality	What it reflects	Exercise-related interpretation	Current evidence level	Trial readiness/key limitations	References
Epigenetic clocks	Horvath DNAmAge, PhenoAge, GrimAge, DunedinPACE	Blood or tissue DNA methylation assays	Accumulated biological age or pace of aging at the molecular level	Habitual exercise/fitness is often associated with slower epigenetic aging; intervention effects are plausible but still heterogeneous across clocks and tissues	Moderate for association studies; emerging-to-moderate for causal intervention inference	Useful as prespecified trial endpoints, but assay cost, tissue specificity, and confounding control remain important	(66, 69, 71, 72, 76, 144)
Inflammation and SASP-related markers	IL-6, TNF-α, CRP, p16INK4a, selected senescence-associated cytokine panels	Plasma/serum cytokines, PBMC assays, targeted molecular panels	Inflammaging, senescent-cell burden, and immune-metabolic signaling	Regular training may reduce chronic inflammatory tone, but acute exercise can transiently raise some cytokines (e.g., muscle-derived IL-6) in adaptive ways	Low-to-moderate for exercise-aging trials because timing and marker selection strongly affect interpretation	Clinically familiar and relatively accessible, but vulnerable to acute illness, medication effects, sampling-time bias, and phenotype ambiguity	(78, 88, 90, 145)
Plasma proteomics and organ-aging signatures	Organ-age proteomic signatures; inflammation-, immune-, and brain-related aging panels	High-throughput plasma proteomics	Multi-organ aging trajectories and pathway-level biological remodeling	May capture broader exercise-responsive remodeling than single analytes and can support organ-specific interpretation	Emerging for exercise-aging applications	High mechanistic value but currently expensive, analytically complex, and less standardized across platforms	(33, 92–94)
Wearable-derived digital biomarkers	Circadian rhythm metrics, gait stability, recovery patterns, PpgAge-like wearable aging clocks	Longitudinal wearable time-series data	Behavioral and physiological resilience measured at high frequency in daily life	Can show how exposure, recovery, and routine regularity relate to later laboratory biomarker change	Emerging	Scalable and non-invasive, but dependent on algorithm validation, device standardization, and careful handling of missing data and privacy	(31, 39, 52, 53)

This revised comparison explicitly distinguishes association-heavy evidence from intervention-ready endpoints. For inflammatory markers, transient exercise-induced changes should be interpreted separately from chronically elevated basal inflammation. For digital biomarkers, current promise exceeds formal validation for anti-aging trials.

## 4 Multisystem mechanisms of exercise in healthy aging

### 4.1 Exercise-induced autophagy and mitochondrial function optimization

**FIGURE 5:**
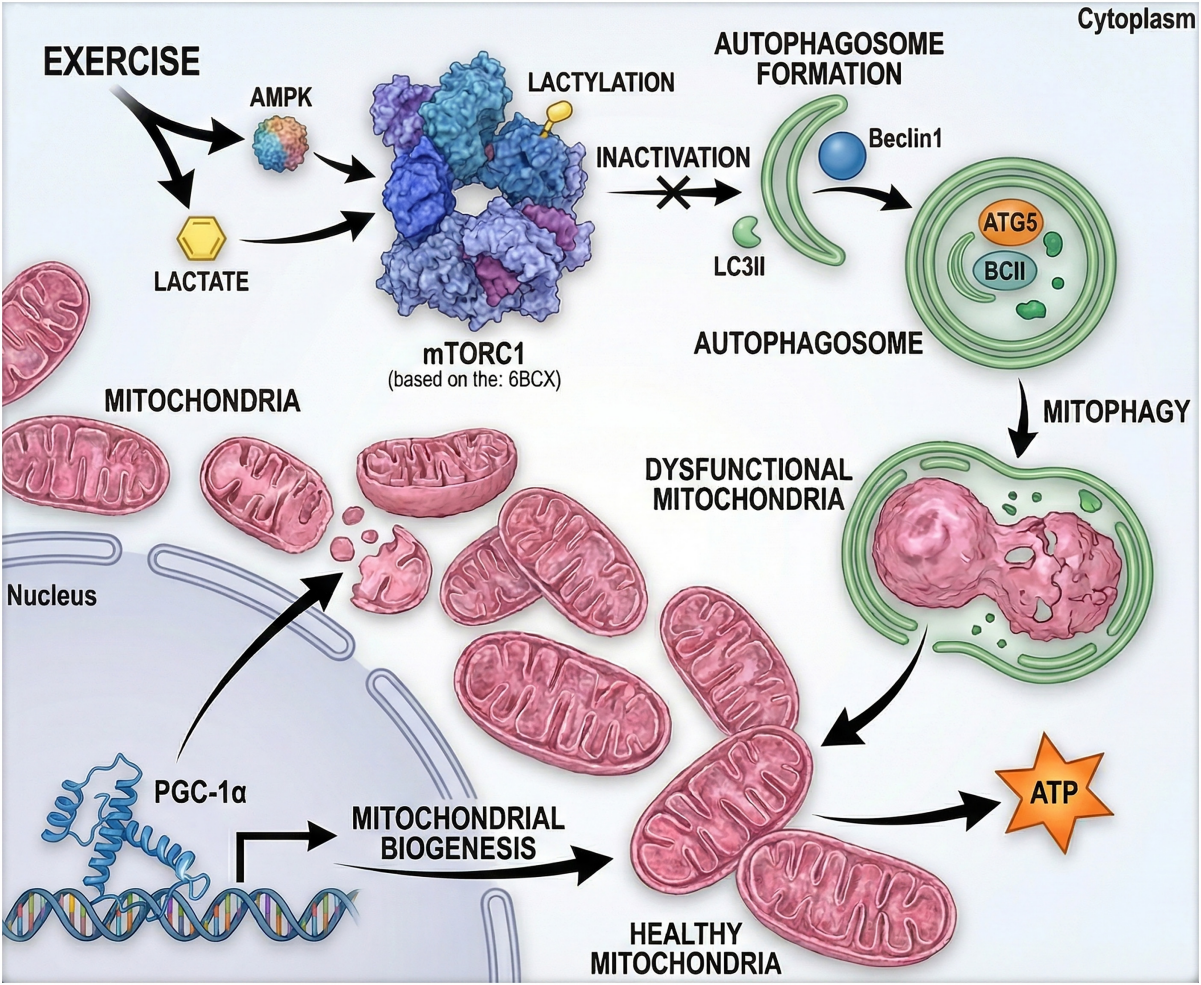
Exercise-induced autophagy–mitochondria crosstalk in aging skeletal muscle. Aerobic exercise elevates lactate, inducing mTOR lactylation (Lys1251) to inhibit mTORC1 and activate autophagy/mitophagy (Beclin1, LC3-II), reducing damaged mitochondria and oxidative stress. In parallel, AMPK–peroxisome proliferator-activated receptor gamma coactivator 1-alpha (PGC-1α) axis signaling promotes mitochondrial biogenesis and oxidative phosphorylation (OXPHOS) assembly.

Exercise is a strong inducer of cellular autophagy, a housekeeping process that maintains homeostasis by recycling damaged proteins and organelles, including mitochondria (101, 102). By enhancing autophagic clearance, exercise may reduce the progressive accumulation of cellular damage that contributes to aging and age-related disease risk (103, 104). Because dysregulated autophagy is linked to sarcopenia, exercise-induced autophagy is also proposed to help preserve muscle integrity by supporting protein turnover and metabolic remodeling (105–107). At the signaling level, exercise commonly promotes autophagy through AMPK activation and relative suppression of mechanistic target of rapamycin (mTOR) activity, facilitating autophagosome formation and lysosomal degradation. Intermittent ischemia-hypoxia exercise preconditioning may induce Beclin1-dependent autophagy, which helps protect cardiomyocytes against exercise-induced myocardial injury (108) (Figure 5). More broadly, exercise can tune autophagic flux in cardiovascular tissue, and maintaining an appropriate flux (rather than simply “more autophagy”) appears important for cardiometabolic resilience (109). In skeletal muscle, lactate generated during exercise has been reported to promote autophagy by stimulating mTOR lactylation, inactivating mTORC1, and thereby supporting autophagic events relevant to mitochondrial quality control and energy regulation (110). Evidence from chronic aerobic training also suggests increases in autophagy markers [e.g., Beclin1, autophagy related 5(ATG5), LC3-II] alongside reduced oxidative stress and inflammation in metabolic tissues (111). Beyond peripheral organs, exercise-related autophagy may extend to the brain, where it can support neuroplasticity and cognitive processes through interactions with brain-derived neurotrophic factor (BDNF) signaling (112). These effects are dose-dependent: insufficient stimulus may yield limited adaptation, whereas excessive training may drive maladaptive autophagic overactivation associated with muscle atrophy and pathological cardiac remodeling (113). In technology-assisted programs, wearable-derived training-load and recovery metrics can help operationalize this dose dependence, aligning prescription with beneficial autophagic windows and enabling more testable links to downstream aging biomarkers in future trials. Altogether, exercise-induced autophagy, together with mitochondrial adaptations, provides a plausible mechanistic bridge between exercise dose and delayed cellular senescence, supporting healthy aging at molecular and systemic levels.

### 4.2 Hemodynamic improvement and enhancement of cerebrovascular function

Exercise has a significant influence on systemic and cerebral hemodynamics, thereby supporting vascular health and cognitive function (114, 115). Exercise improves cerebral blood flow (CBF) by increasing cardiac output and promoting vasodilation, which enhances nutrient delivery and metabolic waste removal in the brain (116, 117). These hemodynamic effects are especially important in older populations, in whom cerebrovascular impairment is a major contributor to cognitive decline and neurodegeneration (118, 119). Moderate activities such as Tai Chi have been shown to improve cerebral blood flow and cognitive function in older adults, highlighting the importance of moderate exercise for brain health (120). Acute interruptions to prolonged sitting, including standing or exercise, may attenuate reductions in total cerebral blood flow. Current evidence does not clearly support uninterrupted sitting as the optimal condition when compared with these alternatives (121, 122). Evidence from advanced imaging methods, such as 4D flow MRI and transcranial Doppler ultrasound (TCD), suggests that exercise-related changes in cerebrovascular reactivity (CVR) and cerebral hemodynamics are associated with favorable brain outcomes, including preserved gray matter volume and improved brain function (123, 124). Exercise also influences cerebrovascular impedance and vascular compliance, both of which are important for maintaining stable cerebral perfusion under changing physiological conditions (125, 126). Physical activity may improve cerebral blood flow dynamics, thereby reducing ischemic risk and supporting recovery in patients with stroke or intracranial atherosclerosis (127, 128). Mechanistically, increased blood flow improves endothelial function and nitric oxide bioavailability, thereby reducing oxidative stress and inflammation, both of which contribute to vascular aging. These vascular benefits improve the delivery of oxygen and nutrients to neural tissue, thereby slowing neurodegeneration and helping preserve cognitive function. Overall, exercise-induced improvements in systemic and cerebral hemodynamics constitute an important component of its anti-aging effects on the brain and vascular system.

### 4.3 Neuroendocrine and immune system regulation

Exercise modulates complex regulatory interactions within the neuroendocrine and immune systems, both of which play important roles in healthy aging and disease protection (129, 130). Exercise increases BDNF, a neurotrophin that supports neuronal survival, synaptic plasticity, and learning and memory (131, 132). BDNF upregulation during exercise can increase neuroplasticity and memory, and autophagy is fundamentally involved in the mediation of this effect (112). At the same time, exercise not only regulates immune responses by enhancing lymphocyte activity, including that of T cells, but also helps maintain immune homeostasis and reduce immunosenescence, the age-related decline in immune competence (113). Hormones of the neuroendocrine system, such as hypothalamic-pituitary-adrenal (HPA) axis, catecholamines, such as noradrenaline, interact with immune cells to control the process of inflammation and stress adaption (133, 134). Indicatively, noradrenaline regulates the oxidative stress of immune cells, resolving the production of reactive oxygen species and antioxidant defense. Exercise-induced changes in neuroendocrine hormones may also influence cytokine production by decreasing pro-inflammatory cytokines such as TNF-α and IL-1β while promoting anti-inflammatory signaling (135). This two-way neuroendocrine-immune system communication promotes tissue homeostasis and resistance to age-related pathologies. Exercise may also influence the neuroendocrine-immune axis through components of the adiponectin receptor system that are involved in autophagy and cellular senescence in skeletal muscle, further illustrating the interconnection of these systems in aging (136). Exercise directly influences neuroendocrine and immune regulation, contributing not only to reduced chronic low-grade inflammation but also to tissue repair and regeneration. These effects support the view that exercise is an essential tool for delaying biological aging and maintaining systemic health.

## 5 Conclusion and perspectives

In summary, technology-assisted physical activity represents an important development in aging research and intervention. It may help delay biological aging by improving exercise adherence and enabling more individualized intervention strategies (42, 137). This review systematically summarizes current evidence on the relationship between technology-facilitated exercise and biological age biomarkers (epigenetic clocks, senescence-associated secretory phenotype or SASP, and organ-specific proteomics), which forms a solid scientific basis to review the effectiveness of exercise interventions on the process of aging.

The biology of aging is complex and systemic, as reflected by the multiple mechanisms through which exercise exerts anti-aging effects, such as regulating autophagy, mitochondrial functioning, hemodynamics, and immune regulation. By examining these pathways, this review highlights the need for a holistic and mechanistically informed approach to the development of exercise regimens that may positively influence the biology of aging.

From multiple research perspectives, it is clear that although conventional exercise interventions are beneficial, technology can enable more precise, adaptive, and scalable physical activity strategies for diverse older populations. Nevertheless, important challenges remain for the clinical translation of these innovations. Major challenges include inequities in technology access among aging populations and the lack of standardized intervention protocols. Addressing these challenges will require the integration of multi-omics data and the development of precision intervention systems that can adapt to individual variability and optimize outcomes. In research settings, Table 1 can be applied by prespecifying sensor-derived adherence/dose metrics and selecting at least one validated aging biomarker class as a key secondary endpoint with standardized baseline and follow-up sampling.

A practical implementation example may help clarify this framework. In a research setting, older adults could undergo baseline profiling that includes functional testing (e.g., gait, balance, and mobility), wearable-derived physiological measures (e.g., heart rate, sleep, recovery, and activity dose), and one prespecified molecular aging panel (for example, an epigenetic clock plus selected inflammatory/SASP markers). Participants could then complete a 12-week wearable- and app-supported exercise program with predefined decision rules for progression, supervision, and safety escalation. At follow-up, biomarker changes would be interpreted together with sensor-derived exposure metrics to distinguish inadequate exercise dose delivery from true biological non-response.

In a clinical or community setting, a lower-cost version of the same framework could rely on consumer wearables, brief remote coaching, and selective biomarker sampling in higher-risk or poor-response individuals. In both settings, implementation will depend on feasibility considerations such as device validity, assay cost, interoperability between wearable and laboratory data systems, missing-data management, clinician workflow burden, and transparent governance for privacy, bias, and algorithmic accountability.

In the future, the integration of artificial intelligence with multimodal intervention strategies may substantially transform technology-assisted exercise paradigms. With AI-based analytics and customizable feedback mechanisms, future interventions may be dynamically optimized to improve effectiveness and adherence. In addition, incorporating these advanced exercise modalities into public health policy may play an important role in achieving large-scale impact and promoting healthy aging at the population level.

To our knowledge, this review is among the first to integrate cross-disciplinary evidence on technology-assisted exercise and biological aging signatures while proposing a “precision exercise anti-aging” framework. Besides philosophical development, this theoretical model can also be used in practical terms to guide potential research and practice in the future. Ultimately, a precision-oriented and integrative perspective will be essential for realizing the full potential of technology-assisted physical activity to slow biological aging, extend healthspan, and improve quality of life in older populations.
